# Linear IgA bullous dermatosis–a fifty year experience of Warsaw Center of bullous diseases

**DOI:** 10.3389/fimmu.2024.1478318

**Published:** 2025-01-14

**Authors:** Cezary Kowalewski, Katarzyna Wozniak

**Affiliations:** Department of Immunodermatology, National Medical Institute of the Ministry of the Interior and Administration, Warsaw, Masovian, Poland

**Keywords:** linear IgA bullous dermatosis, IgA epidermolysis bullosa acquisita, direct immunofluorescence, direct split skin, fluorescence overlay antigen mapping by laser scanning confocal microscopy

## Abstract

Linear IgA bullous dermatosis (LABD) is a rare subepidermal blistering disorder characterized by the presence of linear IgA deposits at the basement membrane zone (BMZ) by direct immunofluorescence (DIF). This entity was first described by Chorzelski and Jablonska from Warsaw Center of Bullous Diseases, Poland. The disease affects children and adults, whereby they differ in terms of clinical picture and course. Among polish patients with LABD mucous membrane involvement was exceptional, although, we reported a case presenting severe scarring of esophagus and conjunctivae with circulating IgG and IgA antibodies to LAD-1 antigen. Severe mucosal involvement was also observed in IgA-epidermolysis bullosa acquisita (EBA). Immunologically, LABD is characterized by circulating IgA antibodies directed to several epitopes of antigen BP180: LAD-1, 97kD, NC16A. Other BMZ antigens, like BP230, laminin 332, type VII collagen or p200 may be affected. We as a first published a case of anti-p200kD pemphigoid mediated by IgA. Our immunoelectron microscopic studies showed that the epitopes recognized by LABD sera are ultrastructurally localized in the lamina lucida. The antigenic heterogeneity, low titer of IgA antibodies and the lack of commercially available tests for some antigens (LAD-1, p200kD) makes the diagnosis challenging in many cases. It is under debate whether these cases are the subtypes of LABD or they represent a separate entities (IgA-p200 pemphigoid, IgA-MMP or IgA-EBA). Since, they differ in terms of clinical course, mucosal involvement, coexisting disorders, response to the treatment and prognosis, their differentiation is mandatory. In the literature there are many cases with undetectable circulating IgA antibodies in whom LABD was recognized based on DIF only. To avoid misdiagnosis, more sophisticated methods should be used, like direct immunoeletron microscopy (IEM), which is a time-consuming technique. The alternative for IEM may be: a) analysis of the BMZ serration pattern, b) immunofluorescence mapping of blister, c) direct salt split (patient’s) skin, d) fluorescence overlay antigen mapping by laser scanning confocal microscopy. The two latter methods were established by the authors years ago and they allowed precise diagnosis (i.e., differentiation LABD from IgA-EBA), initiation of proper therapy and assessment of prognosis in many cases mediated by IgA.

## Introduction

In 1976, Polish scientists Chorzelski and Jablonska reported nine cases in which IgA linear deposits were present at the basement membrane zone (BMZ) and named them “dermatitis herpetiformis and bullous pemphigoid - intermediate and mixed form” (1). Five years earlier, the same group studied the skin of 19 patients with dermatitis herpetiformis (DH) by direct immunofluorescence (DIF) and they found five unusual cases presenting with linear IgA deposits at the BMZ (2). Finally, the name of linear IgA bullous dermatosis (LABD) was proposed by them in 1979 (3).

The distinction between LABD and DH was crucial in terms of the pathomechanism of both diseases, especially given the relation of DH to gluten-sensitive enteropathy, which affects the treatment and prognosis. Further studies have also revealed differences regarding the characteristics of circulating antibodies. Patients with DH presented with IgA anti-endomysial antibodies (4) whereas some LABD patients had IgA anti-BMZ antibodies as determined by indirect immunofluorescence (IIF) (5).

Since the detection of circulating IgA antibodies in LABD using IIF was challenging due to its low serum concentration, it was widely accepted in the 20th century that the diagnosis of LABD could only be established based on the presence of linear IgA deposits in DIF. In the last 50 years, dozens of case reports recognized as LABD by DIF and original papers concerning the subject have been published showing different clinical pictures, prognoses, and responses to treatment.

The implementation of molecular techniques allowed the characterization of target BMZ antigens (6–10). It was found that in the majority of patients, the autoantibodies were directed to bullous pemphigoid (BP) antigens and their epitopes, which corresponded to the localization of IgA deposits in the lamina lucida (11, 12). In these cases, dermal-epidermal separation occurred in the lamina lucida (13–15). However, in some patients, circulating IgA anti-BMZ antibodies were directed to type VII collagen, which is an antigen of epidermolysis bullosa acquisita (EBA). In those patients, IgA deposits were located below the lamina densa, where dermal-epidermal separation occurs in the sublamina densa region (16, 17). Uncommonly, circulating IgA anti-BMZ antibodies may also be directed to other BMZ antigens (p200 antigen, laminin 332) (18–20).

Therefore, it is still a matter of discussion whether LABD is one disorder with a heterogeneous clinical and immunological description (21) or whether the observed cases are examples of different entities mediated by IgA anti-BMZ antibodies (22–25).

Here, we present findings based on the experience of the Warsaw Center for Autoimmune Blistering Disorders and on a review of the literature on the correlation of clinical symptoms with immunological findings in disorders mediated by IgA anti-BMZ antibodies. Due to the lack of commercially available techniques allowing fast and easy detection of IgA anti-BMZ antibodies, the diagnostics of these cases is challenging. Based on the presented findings, we propose a diagnostic management protocol using techniques that enable the localization of IgA deposits within different BMZ structures.

## Epidemiology

LABD is a rare autoimmune bullous disease that most often occurs in adults, with a slightly higher prevalence in women than men (11, 23–25). There is also a pediatric form of this disease that usually has a milder presentation than the adult form. There is a considerable variation in the incidence of LABD between countries, i.e., in Germany, it is 0,25%/million/year (23), whereas in South Korea, it is 4 times higher (26).

The age of the onset of adult LABD differs between Asian and European countries. In Japan and Korea, adult LABD appears between 60 and 65 years of age (26–28), whereas a study performed in Lubeck, Germany on more than 220 LABD cases, indicated an age of onset of over 70 (23). In contrast to Germany, patients in France and Denmark developed LABD at the age of 56 years on average (29, 30), which is similar to Poland. Several reports published in the 20th century by the Warsaw Center of Autoimmune Blistering Disorders on a small number of cases showed that Polish patients with adult LABD were younger than those from Germany (1, 5, 11, 31). Of the 19 well-documented LABD patients described by Wozniak in 2013, four cases were children, and only three out of the 15 adults were aged 70 to 81. The remaining 12 adult patients were aged 29-68 but it is noteworthy that three adult patients were aged 29, 34, and 35 (32). Thus, young patients with LABD seem to be a rather common phenomenon in Poland. Such a difference between Poland, France, and Germany, which are neighboring countries, seems to indicate a need for further analysis.

Pediatric LABD was separated from the chronic bullous dermatosis of childhood (CBDC) group by Chorzelski and Jablonska in 1979 (3). In children, LADB occurs mainly between 1 and 11 years of age and has a peak incidence at 4-5 years (33). It is essential to emphasize that there are also several unusual cases described in the literature as neonatal LABD, which are characterized by severe mucosal involvement and a worse prognosis, unlike in cases of older children with LABD (34, 35).

## Genetics

A British study showed that adult LABD in the Caucasian population was significantly associated with human leucocyte antigen (HLA) Cw7, B8, and DR3 (36), whereas the cases of LABD in children were mostly associated with HLA B8. Additionally, it has been suggested that the presence of the HLA B8 haplotype was associated with a good prognosis (36). In patients expressing the TNF2 haplotype, the duration of LABD appeared to be longer than in those expressing the TNF1 allele (36). In African patients, similarly to Caucasian patients, LABD is also significantly associated with HLA B8, but in Japanese patients that association has not been found (37). In Tunisia, HLADR3 haplotypes were found to be present in 80% of childhood LABD patients (38). In the Chinese population, a Celiac Gene HLA-DQB1∗02:01 seems to be associated with linear IgA bullous dermatosis (39).

Genetic differences between different ethnic groups in LABD may in part explain the different incidences of this disease and the differences in its clinical presentation.

## Pathogenesis

### Target antigens

Circulating IgA anti-BMZ antibodies in patients with autoimmune subepidermal blistering disease (ASBD) are directed to various antigens of the BMZ (**Table 1**). This determines not only the level of dermal-epidermal separation but also the clinical picture and the response to treatment. Both in the cases of childhood and adult LABD, circulating IgA autoantibodies bind to proteolytic products of the extracellular domain of the BP180 antigen, i.e., a 97-kDa protein and/or a 120-kDa protein, which are called LABD97 and LAD-1 respectively (6–8, 50). It is calculated that up to 40% of LABD sera react with full-length BP180 or the NC16A domain of BP180 (9, 10). It has also been observed that some LABD sera concurrently react with several epitopes of the same antigen, which can probably be explained by the intermolecular epitope spreading phenomenon (10). Less frequently, a circulating IgA antibody in LABD binds to an intracellular hemidesmosomal protein—BP230 (43). In some patients, circulating IgA antibodies are accompanied by equally strong IgG anti-BMZ antibodies and they are directed to the same LAD-1 antigen (40). Those patients seem to display a different clinical picture than pure LABD, and they are therefore categorized into a subgroup of so-called LA(G)BD (41, 44, 45, 51–59). Between 5% and 10% of patients with linear IgA deposits in the BMZ present with circulating IgA antibodies directed to type VII collagen—EBA antigen (47, 48). However, it is a matter of debate whether those patients represent the so-called sublamina densa type of LABD (48) or IgA-EBA (22, 23). In Europe, it is postulated that this type of disorder should be named IgA-EBA (25), however, Hashimoto provided a number of historical and immunological arguments supporting the classification of diseases dependent on IgA antibodies as different subtypes of LABD (21). Despite the controversy, there is a general agreement that the above-mentioned cases differ regarding the clinical picture and the response to treatment.

**Table 1:** Target antigens recognized by circulating anti-BMZ antibodies.

References	Antigens recognized by IgA antibodies	Proposed diagnosis
(6, 24, 32, 40, 41)	LAD-1 (fragment of BP180)	LABD
(7, 8)	LABD97 (fragment of BP180)	LABD
(9, 10, 32, 42)	BP180 (full length)	LABD
(9, 10, 32)	BP180-NC16a epitope	LABD
(32)	BP180- C-terminal fragment	LABD
(43)	BP230	LABD
(18, 32, 44–46)	p200 antigen	LABD or Anti-p200 pemphigoid mediated by IgA
(19, 20)	Laminin 332	LABD or anti-laminin 332 MMP
(11, 22, 32, 47–49)	Type VII collagen	IgA-EBA or sublamina densa type of LABD

LABD, linear IgA bullous dermatosis; MMP, mucous membrane pemphigoid; IgA-EBA – epidermolysis bullosa acquisita mediated by IgA.

In rare cases, sera may react with another BMZ antigens, such as p200 (18) or gamma laminin1, as we showed previously, or else with laminin 332 as presented by Hashimoto (20). Therefore, according to the recently published recommendations, the final diagnosis in patients with IgA anti-BMZ antibodies should be based on the characterization of target antigen(s) (25).

### Ultrastructural localization of antigens recognized by IgA anti-BMZ antibodies

In 1995, we used the pre-embedding immunoperoxidase indirect immunoelectron microscopic (IEM) technique to establish the localization of epitopes of the antigens recognized by IgA anti-BMZ antibodies (11).

The study was performed on 27 sera in total: 21 collected from patients with adult LABD, four from those with childhood LABD, and two from patients with MMP mediated by IgA for comparison. Of the 27, 24 sera [19 from the cases of adult LABD, four from those of childhood LABD, and one from the IgA-mucous membrane pemphigoid (MMP-IgA)] reacted on the epidermal side of salt split skin (SSS)-IIF. Those sera reacted in the lamina lucida and/or in the hemidesmosomes on immune electron microscopy (IEM) performed on normal human skin as a substrate. The remaining two sera reacted with the dermal side on SSS-IIF. On the ultrastructural level, they decorated the sublamina densa region, presenting with non-continuous labeling referring to anchoring fibrils. Further studies have revealed that one serum was negative by IB, while the second one reacted with type VII collagen, pointing to the diagnosis of IgA-EBA, not MMP-IgA.

Interestingly, the 24 sera that recognized epitopes of antigen(s) in the upper part of the BMZ presented with four different labeling patterns: a) intercellular labeling of hemidesmosome plaques combined with staining of the underlying portion of the lamina lucida; b) non-continuous labeling of the lamina lucida; c) continuous labeling of the lamina lucida related to the presence of hemidesmosomes; d) mixed labeling pattern of linear lamina lucida and non-continuous lamina lucida and hemidesmosome plaque, suggesting the binding of IgA antibodies to the several epitopes concurrently (11). These findings were further confirmed using an image analysis technique, which made it possible to distinguish specific immune reactions from the background (60).

In the next step, indirect post-embedding immunogold studies were performed on lowicryl K11M-embedded human skin using six selected LABD sera, which strongly reacted with the epidermal side of split skin. All sera recognized antigens localized ultrastructurally in hemidesmosomal plaques and the adjacent lamina lucida. Most of the immunogold particles were found in a linear distribution within the basal cells, directly over the hemidesmosomal portions of the plasma membrane; some extracellular labeling was also noticed (12). In turn, Ishiko et al., using post-embedding immunogold on cryosection and purified IgA anti-BMZ directed against 97kD LABD antigen, found its linear distributions within the upper part of the lamina lucida (61).

Thus, studies conducted using indirect electron immunomicroscopy indicate that IgA anti-BMZ antibodies are directed against various epitopes of the bullous pemphigoid minior antigen 180 (BP180) in LABD patients. Moreover, a mixed labeling pattern, often observed in IEM, suggests that sera from some LABD patients recognize several epitopes of the BP180 concurrently and less frequently; an additional antigen bullous pemphigoid major antigen 230 (BP230) is also present within the hemidesmosomal plaque (11, 12, 60, 61).

### The pathogenic character of IgA antibodies directed to anti-LAD-1 and anti-LABD97

The binding of IgA to a specific Fc alpha receptor, also known as CD89, present on granulocytes has been shown to induce chemotactic migration of those cells to IgA deposits in the skin of LABD patients (62). Blister formation is the result of granulocyte activation and subsequent degranulation, as demonstrated after the passive transfer of an IgA monoclonal antibody to LABD97 antigen in an immunodeficient mouse model (63). The role of granulocyte activation in the pathomechanism of LABD was also demonstrated in further studies on genetically modified mice, which expressed human CD89 after an injection of human IgA anti-BP180 antibodies (64). The separation of the epidermis from the dermis occurs as a result of the activation of proteases, including plasmin and neutrophil elastases, and reactive oxygen species originating from neutrophils, eosinophils, and mast cells (64–66).

Using an *in vitro* LABD model on cryosections of normal human skin treated with anti-BMZ antibodies and then granulocytes, it has been shown that blocking Fc alpha R resulted in inhibition of dermal and epidermal detachment (62). Furthermore, administration of anti-Fc alpha R monoclonal antibodies to a murine LABD model prevented chronic inflammation and tissue damage (62–68). It is worth mentioning that recombinant human IgA1 and IgA2 autoantibodies to type VII collagen induced subepidermal blistering *ex vivo* by a similar mechanism (69).

## Clinical picture of LABD

### LABD in adults

In adults, the skin lesions of LABD are usually polymorphic with associated pruritus. In some cases, the clinical picture is dominated by vesicles, papules, and erythematous patches with a herpetic pattern, resembling DH; however, most patients have large blisters located on the trunk and extremities sitting on inflamed or healthy skin, similar to BP (1, 5, 70, 71). Cases of LABD with a severe clinical picture resembling erythema multiforme, toxic epidermal necrolysis, or erythema annulare centrifugum have also been described (72–76).

The course of LABD in adults is generally chronic with a tendency toward relapses and remissions that last from 6 months to several years; spontaneous remissions occur in approximately 10% of patients (1, 5, 77, 78).

### LABD in children

LABD is the most common autoimmune skin disease in childhood (3, 23, 24, 78–80).

The most common clinical picture of LABD contains blisters and/or vesicles that form a garland-like arrangement, the so-called “clusters” which are most often located on the face around the mouth, in the genital area, and on the hands and feet (3, 78–80). The skin lesions may be accompanied by pruritus. For years, it was thought that such a clinical picture was pathognomonic for children’s LABD, however, recent publications report children presenting with similar characteristics and distribution of skin lesions but being diagnosed with BP (81). Additionally, among our patients, we can report a female pediatric patient with IgA-EBA who initially presented with clusters, suggesting LABD, but a few months later developed milia, typical for EBA (32). Therefore, one should be aware that, at the beginning, ASBDs in children may be indistinguishable. Interestingly, some children with LABD present with erythematous papules or even excoriated plaques resembling allergic disorders. Since the disease usually resolves within 4 years on average and has a good prognosis in the majority of cases, it is classified as a self-limiting disease (3, 23–25, 78–80, 82).

In contrast, LABD in newborns may have an unfavorable course. As has already been reported in publications, the severe course of the disease in this age group was associated with mucous membrane involvement and is usually provoked by drugs (34, 83)—see the section below.

### Mucous membrane involvement

There are significant differences in the literature regarding the occurrence of mucosal lesions in patients with LABD. In the pioneering articles by Chorzelski and Jablonska who described 13 patients with adult LABD (1) and 27 cases of pediatric LABD (3), no mucosal involvement was observed. In subsequent studies from the same center, conducted by the authors of this paper on a well-documented group of 45 patients in whom the recognized antigens and/or ultrastructural localization of IgA deposits at the BMZ were thoroughly described, mucous membrane involvement was found only in three patients. In those three cases, sera recognized type VII collagen in immunoblot or anchoring filaments in the sublamina densa region on IEM (11, 32). Another research group from northern Poland reported 22 LABD cases, which included six patients who presented with mucous membrane involvement (84). Interestingly, eight out of the 22 patients showed reactivity with the dermal side of SSS-IIF, typical for IgA-EBA.

A study performed by a Tunisian research group in a cohort of 31 children showed the occurrence of mucous membrane involvement in 13% (80). A Japanese research group studied 213 cases of LABD and found 26 patients with mucosal lesions, among which 25 were adults and only one was a child. The authors showed that the incidence of mucosal involvement mainly referred to the IgA/G type (27).

In contrast to the above-mentioned papers, a British research group, in their first publication on 25 adult LABD patients and 25 patients with CBDC, found mucosal lesions in 80% and 64% of patients respectively (78). Further studies published by the same authors on a group of 10 patients in whom an LABD diagnosis was based on IgA deposits along the BMZ in DIF performed on the patients’ skin, showed that all of them had oral erosions and 6 also had conjunctival lesions (85). Interestingly, in the biopsies taken from the conjunctiva, only linear IgG deposits were present in DIF, thus conjunctival involvement in those patients depended on IgG, but not on IgA. This observation raises the question of whether such patients present with LABD with mucosal involvement or rather MMP. It is worth noting that conjunctival involvement is the symptom most frequently observed in MMP, whereas in LABD it is not evident. Therefore, such cases require thorough research, especially in terms of target antigens and the precise localization of IgA and IgG deposits.

The vast majority of single cases of LABD described in the literature in which very severe mucosal involvement was shown, including scarring of the esophagus, conjunctivitis, or airway obstruction, were diagnosed based only DIF and histology, without antigen characterization (86–89). However, there are well-documented cases with IgA deposits at the BMZ and conjunctival involvement that in the end fulfilled immunological criteria for IgA-EBA (47, 49, 90, 91).

In turn, we have described a patient with mucosal involvement (critical esophageal stricture and scarring conjunctivitis) in whom circulating IgA and IgG antibodies were directed to the LAD-1 antigen (40). It is still under debate whether such cases should be identified as MMP or IgG/IgA LABD.

Particular attention should be paid to neonatal LABD which occurs in the first weeks, or even days, of life. Only several such cases have been published so far in the form of case reports (34, 83, 92, 93). They are characterized by very severe mucosal lesions, including in the gastrointestinal tract, and sometimes result in fatal outcomes. In all cases but one the diagnosis of LABD was established exclusively based on DIF. Only in one patient was LAD-1 identified as a target antigen, however, in this particular case mucous membranes were not involved and the clinical course was mild and similar to older children (94). Therefore, further research is necessary to understand the pathogenesis of neonatal LABD and to assess the relationship between the target antigen and the clinical course.

In summary, the differentiation in the frequency of mucous membrane involvement in LABD between countries may be a result of ethnic differences but generally is more common in cases of IgA-EBA and LA(G)BD than in the lamina lucida type of LABD.

### Atypical manifestations and course

Single cases of LABD with erythroderma or prurigo nodularis-like lesions have been described (95). The patients presented with nodules instead of blisters, which were accompanied by intense pruritus. Due to an atypical clinical picture, a proper diagnosis was delayed in those cases and they required long-term treatment (96). Atypical LABD may also manifest as different variants of erythema multiforme, including toxic epidermal necrolysis (97). Such reports referred to adults and were usually associated with drug induction, mainly by vancomycin (98).

Atypical cases of LABD may also refer to the course of the disease. We had the opportunity to observe a 3-year-old boy with LABD presenting with vesicobullous eruptions located on the face, around the mouth, on the lower part of the abdomen, and on the genitals, who initially responded well to sulfones and low doses of prednisone. However, despite the systematic treatment, the disease did not disappear and changed its clinical image and course. At the age of 11, the patient developed large, hemorrhagic blisters on the traumatized areas mainly affecting the feet, which resolved leaving scars and milia. Fluorescence overlay antigen mapping using laser scanning confocal microscopy (FOAM-LSCM) performed on the patient’s skin revealed the presence of linear IgA deposits below type IV collagen (32). Analysis of the clinical course of this patient raises the question of whether the sublamina densa type of LABD may not in fact be the same as IgA-EBA.

Another interesting case was initially seen in our department 30 years ago when a 5-year-old boy was admitted with typical manifestations of pediatric LABD mediated by IgA anti-BMZ antibodies directed to the BP180 antigen (32). He responded well to sulfones combined with low doses of prednisone, however, recently, as a 35-year-old man, he visited our department again due to disseminated vesiculobullous lesions resembling a string of pearls. This time, the diagnosis of recurrent LABD was confirmed by DIF. Repeated treatment with disulone in combination with a low dose of prednisone cleared the skin lesions promptly. There is one other case report in the literature of a case of childhood LABD which recurred after puberty (99).

## Associated diseases

### Cancers

A British research group was one of the first to analyze the occurrence of cancer in adult LABD patients. They found nine non-lymphoid and three lymphoid malignant neoplasms in the group of 70 patients. The malignancy rate of non-lymphomatoid neoplasms was almost identical to that which would be expected in an age- and sex-matched population, whereas the frequency of lymphoproliferative diseases among their cases was significantly higher (100). A similar malignancy rate was found in the most recent report on 81 LABD patients. Ten of them had comorbid malignancy (77). Other studies have also confirmed the association of LABD with lymphoproliferative neoplasms (101), most frequently with non-Hodgkin lymphoma (102), chronic lymphocytic leukemia (103, 104), and T-cell lymphoma (105).

LABD may also coexist with visceral malignancy of the urinary bladder (106), esophagus, breast, thyroid gland, and colorectum (28). In most of the above-mentioned cases, the diagnosis of LABD was based only on the presence of linear IgA deposits in DIF.

The retrospective analysis of 58 well-described LABD patients from the city of Kurume, Japan revealed malignancy in 10 of them (28). Other authors from Japan studied a unique group of 32 cases of sublamina densa LABD with a humoral autoimmune IgA response to COL7. In this cohort, they did not find any malignancy (107).

In our center, we also have not noticed any association between LABD and malignancy (11, 32).

The differences in this aspect between countries worldwide might once again be explained by ethnic and genetic diversity. Only an analysis of larger cohorts of patients, well defined in terms of clinical and immunological characterization and subjected to a long follow-up, may lead to a better understanding of the relationship between LABD and malignancies.

### Inflammatory bowel diseases

Kanda et al. reviewed the international literature and reported 35 cases of ulcerative colitis (UC) in patients with well-characterized LABD. The sera of these patients reacted with the roof of IIF-SSS, indicating the localization of those target antigens in the lamina lucida. In the vast majority of those cases, UC preceded the onset of LABD, therefore, the authors suggested a pathogenic relationship between these entities via modification of intestinal antigens due to chronic inflammation, through the production of IgA antibodies and their subsequent cross-reaction with the cutaneous BMZ (108). Several other reports also pointed to an association between LABD and UC (109–111). Less frequently, an association between LABD and Crohn’s disease, another inflammatory bowel disease (IBD), has been reported (112, 113).

However, it has been reported that drugs used in the treatment of UC, such as infliximab, may induce LABD (114). However, in contrast to this finding, we published a case of EBA with coexisting UC, in which infliximab resulted in the long-lasting remission of both diseases (115).

## Agents that provoke LABD

### Drugs

It is worth highlighting that skin eruptions in drug-induced LABD may occur as soon as a few days after the introduction of the offending drug (116, 117). This is opposite to BP or anti-p200 pemphigoid, the diseases mediated by IgG anti-BMZ antibodies directed to the same antigens, in which the time between the introduction of the offending drug and skin lesions development is 6 weeks or even longer (46, 118). It is clear from the point of view of pathology: first, BMZ antigens are modified by the drug and they are then detected by the immune system, leading to the subsequent production of autoantibodies and their binding to the BMZ and followed by chemotactic attachment of leukocytes and enzymatic digestion of the BMZ and blister formation (64). We have described a case of anti-p200 pemphigoid with a clinical picture of Stevens–Johnson syndrome which appeared several days after the introduction of penicillin (46). The DIF performed at the time was negative but when repeated 6 weeks later revealed a strong linear IgG reaction along the BMZ. This particular patient shows that in cases with a clinical picture of AIBD provoked by drugs, DIF may not be positive from the beginning. Therefore, on the basis of our experience in different AIBD, DIF should be repeated with an interval of 4-6 weeks, especially if the skin lesions do not disappear and tend to persist.

In several publications on the subject of drug-induced LABD, skin lesions occurred as early as 2 days after the introduction of the offending drug, but there is no precise information on how long after the appearance of skin lesions the DIF test result was positive (116, 117). Although the current literature lacks satisfyingly comprehensive information on this subject, drug-related LABD may occur soon after the initiation of drug administration, and the immunological mechanism of this phenomenon requires further investigation.

The list of drugs that may cause LABD in both adults and children is long, including antibiotics, non-steroidal anti-inflammatory drugs, antiepileptics, antihypertensive, phenytoin, trimethoprim, immunosuppressive drugs, and even anti-TNF antibody or infliximab (25, 114, 117, 119–122).

It is particularly important to mention vancomycin, which is responsible for more than half of the drug-induced LABD cases (25, 116, 117, 123–126). In most of them, the diagnosis was based on the detection of linear IgA deposits in the BMZ. An interesting study was published by a Japanese research group which proved that circulating anti-BMZ IgA antibodies reacted with vancomycin-modified type VII collagen (123, 127).

From a medical point of view, it is important to note that in some cases of vancomycin-induced LABD, the clinical picture corresponded to TEN with a positive Nikolsky’s sign (124–126). Disruption of type VII collagen in vancomycin-induced LABD suggests the diagnosis of drug-induced IgA-EBA and determines the formation of blisters below the basal lamina, similar to TEN.

In the literature, there are reports on drug-induced LABD with a clinical manifestation of TEN that were caused by drugs other than vancomycin, such as penicillin, phenytoin, diclofenac, and verapamil (25, 123, 128). It should be assumed that the mechanism leading to the formation of those skin lesions is analogous to the mechanism of vancomycin-induced LABD. In such cases, in order to understand the pathogenesis, it is necessary to determine the target antigen recognized by circulating IgA anti BMZ antibodies or alternatively to determine the ultrastructural location of IgA bound in the patient’s skin.

### Vaccinations

Before the COVID-19 pandemic era, cases of vaccine-induced LABD were reported infrequently and mainly referred to children. In these cases, LABD was provoked by vaccines against mumps, measles, or HPV. The time of development of blisters ranged from several days to weeks, similar to drug-induced LADB (129, 130). At that time, only a single case report was published on adult LABD after an influenza vaccination (131).

In the past, the role of vaccines in LABD provocation was thought to be limited to children, however, it seems that they also play a role in adults due to the increase in the use of vaccines against influenza and COVID-19 in this age group (132–134).

### Sunburn and burns

Ten years ago, we described a case of LADB induced by ultraviolet radiation (UV) and discussed five other such cases described in the literature. In three of the six published cases, the diagnosis of LABD was established on the basis of the reactivity of circulating IgA antibodies, with LAD-1 in two cases, and with BP180 in our case, using immunoblotting. In our case, the diagnosis of LABD was additionally supported by FOAM-LSCM showing IgA deposits above laminin 332 and type IV collagen (42). All the patients presented with blisters located on the sun-exposed areas. Among the patients who underwent phototesting, only our case showed hypersensitivity to UVB. The prognosis of LABD induced by UV radiation is good. All patients but one responded well to the treatment and stayed in remission at least for a few years (42).

A case of a 43-year-old Caucasian man with LABD induced by chemical and thermal burns was described but the diagnosis of LABD was based solely on DIF (135).

## Therapy and prognosis

In most LABD cases in children, favorable results are obtained with dapsone in doses of 50-100mg/d (5), during the use of which it is necessary to control the morphology and liver function occasionally. In cases of side effects or contraindications to dapsone, other derivatives of sulfones should be considered, such as sulfapyridine and sulfasalazine (25, 111, 136, 137), which may be better tolerated than dapsone. In isolated cases, it is necessary to add prednisone alongside dapsone to achieve remission (3, 5). Due to the high risk of serious side effects of prednisone in mild cases, local corticosteroids or tacrolimus are worth consideration as a first line of treatment, with or without dapsone or antibiotics (i.e., erythromycin) (138, 139). Cyclosporine has also been suggested as a treatment for severe LABD, however, this drug by itself may induce LABD (119–121, 123, 125, 140).

In cases refractory to dapsone and prednisone, intravenous immunoglobulins or mycophenolate mofetil (23, 25) may be used. If conventional immunosuppressants are not effective, biologicals, such as rituximab or infliximab may be helpful (141–143).

In the treatment of adults with LABD, dapsone at a dose of 100mg/d in monotherapy has been shown to be highly effective, although some patients require higher doses of sulfones (12, 17). In some cases, it is necessary to include low doses of prednisone. In the literature, there are reports on the efficacy of azathioprine, colchicine, methotrexate, tetracycline, or mycophenolate mofetil in LABD (25, 144, 145).

It is not clear why some LABD cases respond to conventional treatment and others require more sophisticated regimens. It has been suggested that it may be in part related to circulating IgA antibodies directed against lamina lucida or sublamina densa antigens (23).

Studies are ongoing on the role of FcαRI as a promising new therapeutic target in LABD (68). Moreover, there is a clinical study in progress in Poland that could lead to the potential registration of anti-CD89 monoclonal antibodies as a treatment for LABD (Eudra CT, number: 2023-508661-33).

## Diagnostics of LABD and other ASBD mediated by IgA anti-BMZ antibodies

### Histology

The histopathological picture of LABD is not uniform (25). In some cases, it shows features characteristic of DH with the presence of clusters of multinucleated granulocytes, arranged mainly in the dermal papillae. In other patients, a subepidermal blister and infiltrates composed of multinucleated granulocytes and eosinophils along the BMZ are observed, similar to BP. In rare cases, the histological picture of LABD combines the phenomena observed in both BP and DH.

### Serology

#### Indirect immunofluorescence

Circulating IgA-anti BMZ autoantibodies in LABD are directed mainly to LAD-1, but also to NC16A of BP180 and BP230, all expressed in the upper part of the BMZ. Therefore, IIF on SSS shows the reactivity of circulating IgA-anti BMZ autoantibodies with the roof of SSS (11, 23, 24, 28, 32, 70, 146). If circulating IgA anti-BMZ antibodies are directed to type VII collagen expressed in the sublamina densa region, they react with the floor of SSS (11, 27, 28, 32, 70, 146). Similarly, circulating IgA or IgG antibodies directed to p200 or laminin 332 antigens also react with the floor of SSS since these antigens are localized in the upper part of the lamina densa (18–20). It is a matter of controversy whether the antibodies that react with the antigens present at the floor of SSS represent a special subset of LABD or are separate entities (21, 25).

Recently, BIOCHIP, a novel diagnostic tool, was introduced for diagnostics of ASBD (146). This is an IIF technique on several substrates in a single incubation field of a laboratory slide including SSS and monkey esophagus for the demonstration of anti-BMZ antibodies and transfected cells expressing the BP230-gC (C-terminal globular domain) recombinant antigens encompassing the NC16a portion of the extracellular domain of BP180 (BP180-NC16a). This technique is mainly used for the detection and characterization of circulating IgG anti-BMZ antibodies, but it is also possible to use it to examine circulating IgA anti-BMZ antibodies, as was recently shown (147).

#### Enzyme-linked immunosorbent assay

Progress in molecular biology has allowed for the synthesis of recombinant antigens, which significantly improved the diagnostics of ASBD. The enzyme-linked immunosorbent assay (ELISA) method, especially multivariant ELISA containing a battery of recombinant antigens (BP180 NC16A, BP230, type VII collagen, envoplakin, and desmogleins 1 and 3) enables the rapid detection of circulating antibodies. The method is characterized by very high specificity and sensitivity (70, 148). Commercially available ELISA is intended for the detection of IgG antibodies. ELISA method kits can also be adapted for the detection of IgA antibodies, however, it requires extensive laboratory experience (70).

#### Western immunoblot

Currently, there is no commercially available test allowing for the detection of IgA circulating antibodies to LAD1/LABD97 and LABD antigens. The detection of these antigens is enabled by immunoblot and is possible only in a few laboratories around the world (7–10, 24, 52, 70). Thus, the detection of IgA anti-BMZ antibodies is challenging even if using a battery of methods and the detection rate ranges from 20% to 75%, depending on the cohorts of studied patients, technical capabilities, and experience of diagnostic laboratories (24, 70, 79, 80).

### Direct immunofluorescence

Direct immunofluorescence performed in a patient’s skin shows the presence of linear IgA deposits along the BMZ in all or nearly all patients, therefore, in cases with negative DIF but clinical characteristics of LABD, the biopsy should be repeated (23, 25). A study published by Becker disclosed only one patient out of 220 examined LABD cases was negative in DIF (23).

The fluorescence pattern of IgA in DIF is varied; in some patients it is thin and linear, but more often it forms a thick, linear, or even fibrillar staining, therefore the assessment of DIF in LABD may sometimes lead to confusion with granular staining, especially when observed under low magnification (2, 3, 149). It is likely that this characteristic thick band is the result of the concurrent reactivity of IgA anti-BMZ antibodies with different epitopes of BP180 and BP230 as presented by Kowalewski with the use of indirect IEM (11, 60). Interestingly, DIF stays positive in LABD from the onset of the disease, during the entire treatment and for many years after the therapy was finished (5). Therefore, in contrast to pemphigus and pemphigoid, positive DIF in the remission of LABD should not be, in our opinion, recommended as a decision criterion for continuing or ending the therapy.

In the majority of LABD patients, IgA in the BMZ is a single component but in 30% of the cases, it can be accompanied by C3 linear deposits (1–3, 5). In some of the patients, IgG linear deposits could also be detected (1, 2, 5, 23, 25, 28). If IgG is equally as strong as IgA, the diagnosis of LA(G)BD has been suggested by Hashimoto (41, 43), and it would be a diagnosis of an entity overlapping with LABD and pemphigoid in terms of clinical and immunological features (23, 40, 52).

Linear IgA deposits observed in DIF are not a hallmark for the diagnosis of LABD, since IgA deposits along the BMZ are also observed in IgA-EBA (22, 23, 32, 47) and anti-p200 pemphigoid (18, 44). Thus, in each case the diagnostics process should be supported by other techniques, allowing for the identification of the target antigen or for precise localization of IgA deposits in the BMZ.

### Techniques allowing precise localization of IgA deposits in the BMZ

#### Serration pattern of linear IgA deposits

More than 20 years ago, Vodegel et al. introduced a method for differentiating BP from EBA based on the serration immunofluorescence pattern of IgG deposits in the patient’s skin. Subsequently, the same research group and others confirmed the possibility of distinguishing between IgA deposits in the BMZ using the same methodology (150). It has been shown that an n-serrated pattern corresponded to the localization of either IgG or IgA deposits in the hemidesmosomes, lamina lucida, or lamina densa on the ultrastructure level, whereas an u-serrated pattern referred to the ultralocalization of type VII collagen in the sublamina densa (25, 70, 146, 150).

The analysis of n- and u-serrated patterns requires high-quality IF slides, a high-resolution lens, and extensive experience of the IF reader(s) (150). In cases where thick or fibrous IF staining is present, the analysis of the serration pattern is more challenging compared to BP or EBA. At the moment, a multi-center European program (the MAXISPA study), the objective of which is to research this method, is being conducted under the EADV grant to improve the ability in diagnostics of SBD.

#### Immunofluorescence mapping of lesioned patient’s skin

Immunofluorescence mapping (IFM) on a patient’s blister is a widely recognized diagnostic method, originally described for the differentiation of hereditary epidermolysis bullosa (151). The method allows the localization of blister formation using appropriate BMZ markers (i.e., antibodies against laminin 332 as a marker of the lamina lucida-lamina densa border and/or type IV collagen as a marker of lamina densa (151).

The same method may be used to determine the location of blister formation in patients with ASBD mediated by IgA. The presence of both markers in the floor of the blister indicates separation of the epidermis from the dermis within the lamina lucida (lamina lucida type of LABD), whereas the presence of both reactants in its roof indicates separation below the lamina densa (sublamina densa type of LABD or IgA-EBA).

The advantage of the IFM technique is the possibility to perform retrospective analyses of paraffin-embedded tissues in search of immunostaining with type IV collagen antibody (152). Diagnostics using IFM are limited to the patients presenting with fresh blisters.

#### Direct salt split skin performed in patients’ own tissue

Direct salt split skin (DSSS) may be helpful for diagnosis in cases with undetectable circulating antibodies. DSSS was originally described by Kowalewski in his doctoral thesis in 1989 (153) and subsequently by Gammon and Kowalewski in 1990 (154). Originally, the authors showed the presence of IgG deposits in the epidermal side of the blister or in both epidermal and dermal sides in BP, which corresponded to its ultrastructural localization in the entire lamina lucida. Whereas in EBA, IgG deposits were located at the bottom of the artificial blister, which corresponded to its ultrastructural localization in the sublamina densa (154).

**Figure 1:**
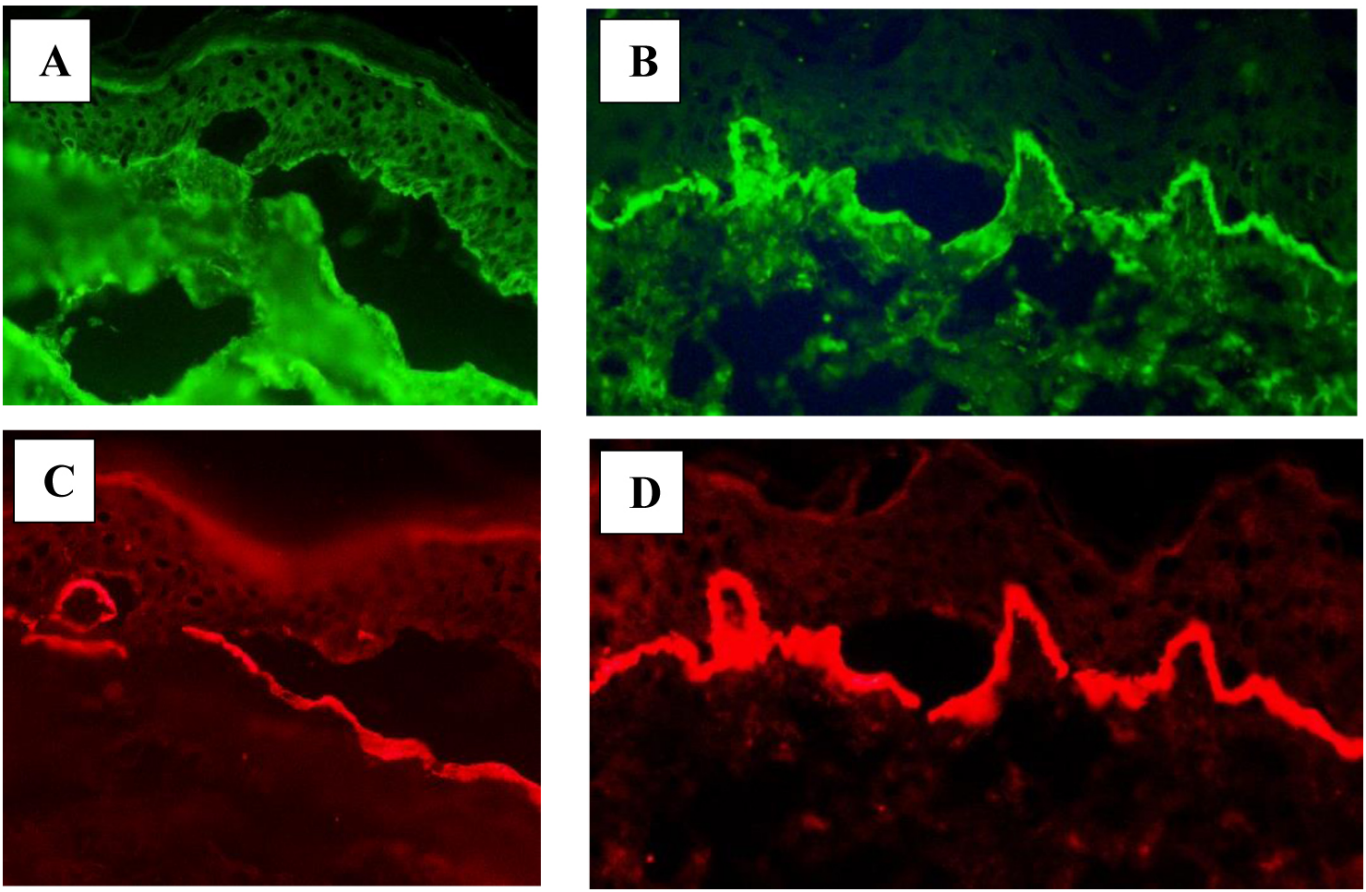
Direct salt split skin. **(A)** IgA deposits (green) located on the epidermal side of the blister in a patient with LABD. **(B)** IgA deposits (green) located on the dermal side of the blister in a patient with IgA-EBA. Antibodies against laminin 332 (red) proving dermal-epidermal separation in the lamina lucida located on the dermal side of the blister in LABD **(C)** and IgA-EBA **(D)**.

In LABD, IgA deposits were located on the epidermal side of the separation (153). Here, we present a case of LABD in which IgA deposits were located on the epidermal side of the artificial blister (**Figure 1A**) and a case of IgA-EBA with linear IgA deposits at the bottom of the blister in DSSS (**Figure 1B**). The blister formation within lamina lucida in DSSS was confirmed by dermal staining of laminin 332 antibodies using IFM (**Figures 1C, D**).

In 2019, a French group studied two patients with linear IgA deposits on DSSS and found these deposits on the epidermal side of the blister in one case and on the dermal side of the blister in the second patient (142). Thus, DSSS allows one to distinguish between IgA deposits located in the lamina lucida and those located in the sublamina densa (142, 153). The great advantage of DSSS is that it is easy to apply in all patients. The limitation of the method is that dermal pattern has to be differentiated from those present in anti-p200 pemphigoid and anti-laminin 332 pemphigoid mediated by IgA.

### Ultrastructural localization of IgA deposits at the BMZ

#### Direct immunoelectron microscopy

Direct immunoelectron microscopy using the pre-embedding immunoperoxidase technique (DIEM) performed on skin biopsies from patients with linear IgA deposits in the BMZ, showed the presence of IgA deposits in the lamina lucida of the BMZ in the majority of cases (13–15). Less frequently, IgA deposits were found in the sublamina densa region (16, 17).

Interestingly, there is only one study revealing a so-called “mirror image pattern”, referring to the concurrent localization of IgA deposits in the lamina lucida and sublamina densa (155). Thus far, the nature of this phenomenon has not been explained.

Though IEM has a high resolution, it is not a routine technique in the diagnostics of ASBD due to the time-consuming nature and demanding procedure.

### Fluorescence overlay antigen mapping with the use of laser scanning confocal microscopy

Alternatively to IEM, in 2003, we developed a method named fluorescence overlay antigen mapping using laser scanning confocal microscopy for practical differentiation of subepidermal bullous diseases mediated by IgG anti-BMZ antibodies (BP, MMP and EBA) (156). This is a method of choice for cases in which circulating anti-BMZ antibodies are not detectable and characterization of the target antigens is impossible. Originally, we compared the localization of linear IgG deposits to the localization of different BMZ markers: laminin 332 and type IV collagen (156). Our study disclosed that, in BP, the patients’ IgG deposits were located above type IV collagen and laminin 332 whereas in EBA, IgG deposits were localized below type IV collagen. In MMP patients, IgG deposits were located between laminin 332 and type IV collagen (156).

**Figure 2:**
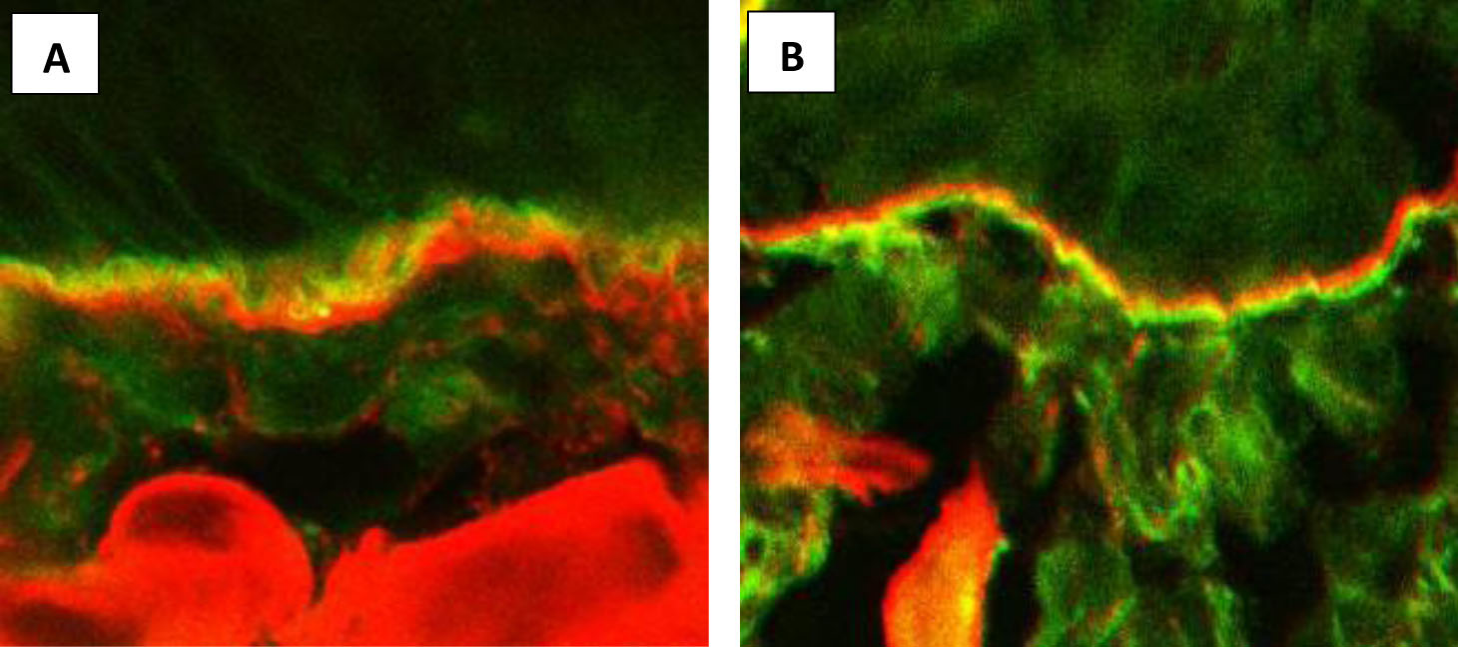
Fluorescence overlay antigen mapping by laser scanning confocal microscopy. **(A)** IgA deposits (green) located above type IV collagen (red) in a patient with LABD. **(B)** IgA deposits (green) located below type IV collagen (red) in a patient with IgA-EBA.

In 2013, we applied this method for the differentiation of the diseases mediated by IgA anti-BMZ. FOAM-LSCM was performed in 19 patients with disseminated tense blisters, who presented with *in vivo* bound and circulating IgA anti-BMZ in immunofluorescence tests (32). FOAM-LSCM disclosed IgA deposits above type IV collagen in 14 of the 19 cases, characteristically of the lamina lucida type LABD, whereas in the remaining five patients, IgA deposits were located below type IV collagen, suggestive of sublamina densa LABD or IgA-EBA (**Figure 2**). FOAM-LSCM studies were supplemented by immunoblotting showing that IgA antibodies in 11 of the 14 patients with deposits above type IV collagen reacted with different epitopes of BP180, but mainly with LAD-1, which is the target antigen in LABD. In one patient with IgA, deposits above type IV collagen serum reacted with the 200kD antigen. Among the five patients with deposits below type IV collagen, one had antibodies to the 290-kDa type VII collagen by immunoblot, whereas another three patients were positive with recombinant type VII collagen by ELISA (32).

It is worth mentioning that, thanks to FOAM-LSCM, we were able to diagnose anti-p200 pemphigoid mediated by IgA on the basis of co-localization of IgA deposits with laminin 332 (18). The results of our research have proven that it is possible to differentiate the lamina lucida type of LABD from the sublamina densa type of LABD and IgA-EBA.

It is also possible to assess the binding site of circulating IgA antibodies in the BMZ using FOAM-LSCM, if characterization of target antigens has failed (157, 158).

## Conclusions

Linear IgA deposits in the BMZ detected by DIF are not pathognomonic for LABD and may also be present in other ASBD mediated by IgA-anti BMZ antibodies. These diseases differ in terms of clinical course and response to treatment even though they may present clinical similarities at the onset of the disease. In LABD, circulating antibodies are directed to various epitopes of the BP180 antigen located in the hemidesmosomes and lamina lucida. For practicing dermatologists, it is very important to distinguish LABD from other diseases in which IgA antibodies recognize antigens of the lower part of the BMZ—mainly type VII collagen, characteristic in IgA-EBA, and less frequent diseases such as IgA-anti p200 pemphigoid. The final diagnosis should be established on the basis of the clinical picture and the characteristics of the target antigen(s), if possible.

However, in cases in which circulating antibodies are not detectable, it is mandatory to establish the location of IgA deposits in the patient’s skin using accessible methods, i.e., immunofluorescence mapping of the patient’s lesioned skin, serration immunofluorescence pattern, or direct split of patient’s skin, which clarifies the diagnosis in almost all cases, and FOAM-LSCM, which can precisely distinguish LABD from IgA-EBA.
